# After diagnosis, place matters: the role of neighborhood built environment in aging in place among older adults with cognitive impairment

**DOI:** 10.1093/geronb/gbag057

**Published:** 2026-04-03

**Authors:** Wenjin Wang, Chanam Lee, Xi Chen, Xuemei Zhu, Marcia G Ory

**Affiliations:** Department of Landscape Architecture and Urban Planning, Texas A&M University, College Station, Texas, United States; Department of Landscape Architecture and Urban Planning, Texas A&M University, College Station, Texas, United States; Department of Landscape Architecture and Urban Planning, Texas A&M University, College Station, Texas, United States; Department of Architecture, Texas A&M University, College Station, Texas, United States; Department of Environmental and Occupational Health, School of Public Health, Texas A&M University, College Station, Texas, United States; (Social Sciences Section)

**Keywords:** Neighborhood, Dementia, Mixed-methods, Dementia-friendly community, Environmental perception

## Abstract

**Objectives:**

As populations age, dementia is becoming increasingly prevalent, posing major public health challenges. Aging in place (AIP) is an important policy and personal objective for older adults, including those experiencing cognitive impairment. We investigated whether specific environmental features influence AIP of older adults with cognitive impairment and how cognitive impairments or dementia diagnoses affect caregivers’ environmental perceptions and decision-making.

**Methods:**

This convergent mixed-methods study surveyed 95 caregivers of older adults with cognitive impairment. Logistic regression was used to identify neighborhood features that predicted the AIP outcome, while thematic analysis examined caregivers’ qualitative narratives on how neighborhood features shape AIP decisions.

**Results:**

Neighborhood features had limited direct effects on the AIP outcome, regardless of dementia severity. However, a significant interaction emerged between formal diagnosis and perceived safety (*p* < .01). This indicates an important moderating role of diagnosis status, with each additional point on the safety scale associated with a 10% increase in the AIP odds among those with a formal dementia diagnosis. Qualitative findings reinforced the importance of perceived safety, while also highlighting accessible destinations/services and community support as additional facilitators of AIP.

**Discussion:**

These findings suggest environmental features and diagnostic status jointly shape AIP decisions. A timely dementia diagnosis may heighten caregiver awareness of environmental supports and barriers, enabling informed adjustments. To create inclusive neighborhoods that support AIP, coordinated approaches integrating early diagnosis, dementia-friendly design, and community support are needed. Such approaches can contribute to reducing institutional transitions and caregiver burdens.

The global population is aging rapidly. Cognitive decline is a common part of aging, often beginning with mild impairment and progressing to an increased risk of Alzheimer’s disease (Murman, 2015; National Institute on Aging, 2023). Dementia, with Alzheimer’s disease as its most common form, has emerged as a major public health concern (World Health Organization [WHO], 2025). In the United States, approximately 7.2 million Americans aged 65 and older are living with Alzheimer’s dementia (Alzheimer’s Association, 2025). Aging in place (AIP) has been a long-standing goal on both personal and policy levels. Globally, AIP is widely promoted by governments and professional organizations. Broadly described as “the ability of older people to live in their own home and community safely, independently, and comfortably, regardless of age, income or level of intrinsic capacity” (WHO, 2015, p. 36), AIP emphasizes an ideal of supporting independence and well-being in older age. Meanwhile, this ideal also reflects a reality that approximately 85% of older adults with dementia in the United States reside in community settings rather than institutional facilities (Chi et al., 2019).

Although AIP is increasingly advocated and practiced, current literature offers no clear consensus on its definition (Bigonnesse & Chaudhury, 2020). Common understandings range from place-based definitions (e.g., never moving, staying put as long as possible, staying in the same vicinity) to service-based approaches (e.g., staying out of nursing homes, not moving between aged care facilities) and more flexible control-based definitions that emphasize autonomy, choice, and diverse residential pathways (Forsyth & Molinsky, 2021). These varying definitions are rooted in distinct theoretical perspectives (Bigonnesse & Chaudhury, 2020; Forsyth & Molinsky, 2021). From a functionalist or biomedical perspective, AIP emphasizes maintaining independence by modifying physical environments to accommodate declining capacities. A structuralist and critical perspective instead highlights how social and economic structures shape aging choices, emphasizing empowerment, service access, and inclusion. The phenomenological perspective centers on lived experiences, including belonging, place attachment, and identity. Ecological or systemic frameworks emphasize the dynamic fit between personal capacities, environmental conditions, and available supports. These conceptual variations directly affect how AIP is measured, interpreted, and supported across different populations and policy settings.

For older adults experiencing cognitive impairment, AIP remains a critical but more complex goal. As cognitive and functional capacities decline, they increasingly rely on their immediate surroundings to maintain daily independence, access essential services, and sustain social connections (Lawton, 1983). Therefore, the neighborhood plays an important role in supporting their social health and well-being (Clark et al., 2020). Traditional, place-based approaches define AIP as the intention to remain in one’s current home or neighborhood rather than relocate to a senior living facility (Bigonnesse & Chaudhury, 2020, p. 235). Broadly referring to this definition, this study centers on the role of neighborhood environments in AIP among older adults with cognitive impairment.

## Literature review

Recognizing the role of environment in supporting aging populations, the WHO’s Global Age-Friendly Cities Guide (n.d.) proposed eight key domains, including outdoor spaces and buildings, transportation, housing, social participation, respect and social inclusion, civic participation and employment, communication and information, and community and health care, that together shape an age-friendly environment. However, the guide offers limited guidance for addressing the specific needs of those with cognitive impairment.

In response, the dementia-friendly initiatives emerged, emphasizing the importance of creating supportive environments where people with dementia are understood, respected, and supported (Wu et al., 2019). These initiatives connect closely with broader discussions of AIP for older adults with cognitive impairment. Prior literature identifies a wide range of factors influencing AIP for the general aging population, including individual characteristics (e.g., income, migration history), social support and interactions (e.g., community ties), community-based services (e.g., service availability, assisted housing), mobility, and built environment (e.g., housing satisfaction, environmental stress) (Bigonnesse & Chaudhury, 2020; Forsyth & Molinsky, 2021). To meaningfully support older adults with cognitive impairment, these factors must be adapted to the evolving challenges associated with cognitive decline.

Built environment plays a particularly foundational role among the factors influencing AIP. Emerging research emphasizes the critical role of the built environment in shaping the daily experiences of people with dementia (Gan et al., 2022). Key pillars such as Mitchell et al.’s (2010) six design principles have significantly influenced the design and evaluation of dementia-friendly neighborhood environments. Accessibility refers to enabling people to reach, enter, and use spaces despite physical, sensory, or cognitive impairments. Legibility and distinctiveness support orientation and wayfinding through clear layouts and recognizable cues. Safety focuses on allowing people to move around without fear of harm. Familiarity supports recognition and understanding of surroundings, aiding memory and orientation. Comfort refers to the ability to use and enjoy places without physical or psychological discomfort.

While these principles provide valuable guidance, understanding their impact requires attention not only to the design of physical features, but also to how older adults perceive, experience, and interact with the environment (Wahl et al., 2012). For those with cognitive impairment, the influence of the environment on AIP is also shaped, perhaps even more so, by personal and contextual factors. Older adults with cognitive impairment often appear less aware of how the built environment affects their daily lives (Biglieri & Dean, 2021; Mitchell & Burton, 2006; Mitchell et al., 2004). This lack of awareness may stem from individuals increasingly attributing their difficulties in daily lives to personal deficits rather than environmental factors (Biglieri & Dean, 2021; Mitchell & Burton, 2006). Furthermore, the absence of a formal diagnosis may also limit awareness, as both individuals with cognitive impairment and their caregivers may fail to recognize early symptoms and behavioral changes or dismiss them as unremarkable or not requiring adjustment (Perry-Young et al., 2018; Stokes et al., 2015; Waymouth et al., 2023). These dynamics suggest that the severity of cognitive decline and diagnosis-related status may play important roles in shaping how environmental factors are perceived, and ultimately, affect AIP outcomes.

Together, the environmental and personal dimensions shape not only the feasibility of AIP but also the pathways through which support can be appropriately aligned with cognitive needs.

## Research objectives

Previous studies have examined how built environments influence the behavior, function, and well-being of people with cognitive impairment, with implications for their AIP (Chen et al., 2022; Gan et al., 2022; Sturge et al., 2021). However, relatively little attention has been paid to whether these outcomes translate into actual AIP outcomes of older adults with cognitive impairment, and even fewer have considered how the personal and contextual factors may condition such relationships. Framed within an ecological perspective, this study conceptualizes AIP as an outcome of dynamic interactions between individuals and their environments. By focusing on the older adults with cognitive impairment, this study aims to address the aforementioned gaps by investigating the role of neighborhood built environments in their AIP outcomes and the moderating influence of cognitive condition and diagnosis status.

Such insight is essential for the planning and design of future communities, as well as improving existing ones to be more inclusive and supportive for older adults with cognitive impairment. These efforts can benefit those and their caregivers by reducing the relocation challenges and easing care burdens. Furthermore, they can benefit our society by lowering the costs associated with institutional care.

## Methodology

### Study design

**Figure 1:**
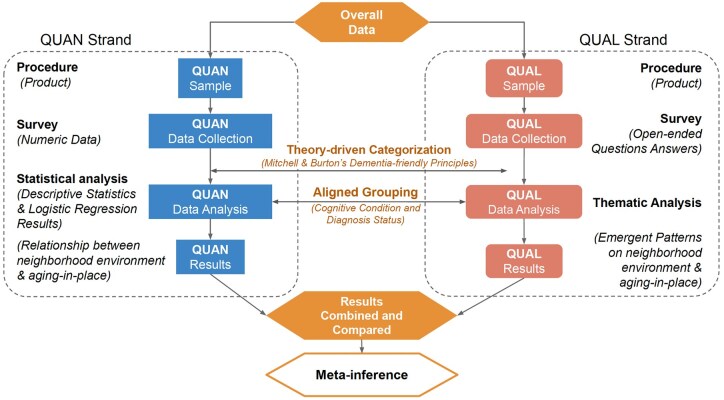
Convergent quantitative (QUAN) and qualitative (QUAL) mixed-method design.

This study was part of a larger five-year research project “*Active Living Austin (ALA)*” that explored the impacts of moving to an activity-friendly community on residents’ physical activity and health, funded by the U.S. National Institutes of Health (NIH R01CA197761). It draws on data from an ancillary study, *Dementia-Friendly Communities to Promote Active Living in Persons with Alzheimer’s disease and related dementias (AD/ADRD)*. We used a convergent mixed-methods design to capture both statistical trends and contextual understanding (see Figure 1). The quantitative and qualitative data were collected in parallel with a single survey collecting closed-ended items and open-ended text, but analyzed independently. While the two strands differed in data type, analytic approach, and resulting products, integration was planned through a shared theoretical framework, aligned grouping, and final side-by-side comparison to generate meta-inferences. These integration steps are described in more detail later.

This study design was informed by the pragmatism paradigm, emphasizing practical outcomes and methodological flexibility to address complex, real-world problems (Creswell & Clark, 2018). It supported the use of mixed-methods by focusing on what works to generate actionable insights and enabled meaningful integration of quantitative and qualitative data. This approach was particularly relevant to our aim of informing more inclusive and supportive community design for older adults with cognitive impairment.

The purpose of the quantitative strand was to test two hypotheses: (a) more dementia-friendly neighborhood built environmental features make older adults with cognitive impairment more likely to age in place rather than relocate; and (b) cognitive condition and diagnosis status amplify the relationship between neighborhood built environment and the AIP outcome. Proxy (caregiver) reported data were collected using structured survey questions that measured perceived neighborhood features, relocation intention, cognitive condition and diagnosis status, and demographic and other potentially relevant information. The qualitative strand enabled a deeper and more direct understanding of how perceived neighborhood factors either support or hinder AIP for individuals with cognitive impairment. This was explored through open-ended survey questions, allowing respondents to describe environmental challenges and facilitators in their own words with their lived experiences.

The rationale for integrating the quantitative and qualitative strands was triangulation. Specifically, this involved corroborating the findings from the quantitative analysis with insights from the qualitative data and identifying areas of convergence and divergence to address our overarching mixed-method research question: *how the neighborhood environment influences the AIP outcome for older adults with cognitive impairment.*

### Study sample and recruitment

This study adopted a proxy-respondent approach. Survey data were collected between August 2020 and June 2021 from family caregivers who had provided care to an individual aged 50 or older living in the community with noticeable cognitive decline severe enough to interfere with everyday activities. Older adults are generally less likely to participate in surveys and may provide incomplete or unreliable responses (Neumann et al., 2000). These challenges are more pronounced for those with cognitive impairment, who often struggle to articulate their preferences or needs (van Der Roest et al., 2007). As a result, proxy respondents have long played a critical role in aging and dementia research (Arons et al., 2013; Neumann et al., 2000). Using family caregivers as key informants ensured feasibility and ecological validity and addressed ethical and practical constraints. Eligible participants must (a) have been providing direct caregiving assistance to the person with memory problems for at least 10 hr per week during most weeks beginning no later than February 1, 2020 and (b) be familiar with both the daily routine and the surrounding community of their care recipients.

We recruited caregivers through diverse channels, including state and local agencies, nonprofit organizations, age-targeted residential communities, and university-affiliated networks. Outreach methods consisted of flyers, email campaigns, newsletters, and social media announcements. This study was reviewed and approved by the Texas A&M University Institutional Review Board.

### Data analysis

#### Quantitative data analysis

##### AIP outcome variable

This study adopts a place-based operationalization, consistent with the traditional understanding of AIP, defined as continued residence in a private home or community setting rather than a transition into institutional senior care (Bigonnesse & Chaudhury, 2020, p. 235; Forsyth & Molinsky, 2021). AIP is measured using a functional proxy based on caregiver responses to the binary-coded question (referring to the pre-COVID period): *Has this person (the one with cognitive impairment) planned to move into a senior living facility (e.g., assisted living, nursing home, retirement community) in the future*? Care recipients who had no plans to move into a senior living facility were classified as AIP (=1), while those who expressed an intention to relocate were coded as non-AIP (=0). This operationalization is consistent with previous research measuring AIP intentions and residential stability among older adults (Battisto, 2004; Choi, 2022).

##### Perceived neighborhood environment variables

We assessed neighborhood environments by asking caregivers to report the presence or absence of specific features in the care recipients’ neighborhoods with reference to conditions before COVID-19. Given our study’s focus on the built environment, a total of 40 items were categorized within a theory-driven framework of key dementia-friendly environmental domains (i.e., safety, accessibility, legibility/distinctiveness, comfort) (Mitchell & Burton, 2010). Legibility and distinctiveness were combined as both reflected related aspects of wayfinding and spatial orientation, while familiarity was excluded from quantitative analysis due to its highly subjective and context-dependent nature. A detailed breakdown of items is provided in Table S1. Response options included yes, no, and not sure. Each item was scored based on caregiver responses: yes coded as 1, no as −1, and not sure as 0, with reverse coding applied to negative characteristics (e.g., high crime rate). While individual features may differ in impact, we applied an equal-weight approach to avoid subjective assumptions about their relative importance.

##### Cognitive condition and diagnosis status variables

Cognitive impairment was assessed by the Quick Dementia Rating System (QDRS), a validated tool for distinguishing dementia presence and staging (Galvin, 2015). QDRS estimated both the likelihood and severity of dementia by evaluating 10 domains (e.g., memory, orientation, and daily functioning) with total scores ranging from 0 to 30. Based on established thresholds, cognitive status was categorized as normal cognition (0 ≤ QDRS ≤ 1.5), mild cognitive impairment (2 ≤ QDRS ≤ 5.5), mild dementia (6 ≤ QDRS ≤12.5), moderate dementia (13 ≤ QDRS ≤20.5), and severe dementia (21 ≤ QDRS ≤ 30). Diagnosis status was measured using a binary variable based on caregiver responses to whether a healthcare provider had ever diagnosed the individual with Alzheimer’s disease or any type of dementia.

##### Statistical analysis

We conducted statistical analyses using Stata 18, and statistical significance was determined as *p* ≤ .05. We first conducted descriptive analyses to examine the characteristics of older adults with cognitive impairment and neighborhood environment characteristics reported by their caregivers. Given the binary nature of the AIP outcome variable, we performed multivariable logistic regression analyses. The base model included only the primary variables of interest: neighborhood built environment domains. We then incorporated confounding variables representing individual-level characteristics, such as demographic attributes and living conditions. Finally, to examine whether cognitive condition moderated the relationship between neighborhood built environment and the AIP outcome, we estimated two separate interaction models: one testing the interactions between QDRS score and the built environment variables to assess the role of impairment severity, and the other testing interactions with formal diagnosis to evaluate the effect of dementia diagnosis. This sequential modeling approach enabled us to explore both main effects and conditional relationships with the modest sample size.

#### Qualitative data analysis

We followed Braun and Clarke’s thematic analysis framework (Braun & Clarke, 2012): (1) familiarizing ourselves with the data, (2) generating initial codes, (3) searching for themes, (4) reviewing potential themes, (5) defining and naming themes, and (6) producing the report to analyze responses from two open-ended questions: *In general, what aspects of one’s neighborhood make it DIFFICULT (or EASIER) to age in place (staying in their own community without having to move to a care facility) for people with dementia and/or severe memory problems?* Specifically, in addition to identifying data-driven patterns, we also used the established six dementia-friendly environmental principles (Mitchell & Burton, 2010) to inform and structure our coding. For neighborhood features less directly related to the built environment but represented distinctive patterns, we used the WHO Age-Friendly Cities framework’s (n.d.) eight domains mentioned earlier to guide their categorization. To facilitate comparative interpretation, each participant was labeled by their cognitive condition based on the QDRS score and formal dementia diagnosis. One researcher led the coding and synthesis of results, and then presented them to the team for discussion and resolution of any discrepancies.

#### Mixed-methods integration procedures

Integration occurred at multiple points in the research process to strengthen interpretive validity and offer a comprehensive understanding (Bazeley, 2024). We adopted a connecting design, linking the quantitative and qualitative data at the theoretical and sampling stages (Fetters et al., 2013). Specifically, the coding structure used in the qualitative analysis and the categorization of quantitative neighborhood characteristics were both informed by the same theoretical framework (Mitchell & Burton, 2010). Both quantitative and qualitative analyses considered cognitive condition and diagnosis status as key grouping variables for between-group comparisons. We further incorporated merging by bringing the two strands together at the interpretation stage to generate meta-inferences following the triangulation rationale described previously (Fetters et al., 2013). After independently analyzing the two strands, findings from each strand were systematically compared and contrasted with a joint display. This joint display visually represented side-by-side alignment of the statistical trends and the key themes, organized by cognitive condition and/or diagnosis status.

The intent of this integration process was primarily to corroborate findings across the two strands, emphasizing convergence and divergence. However, if the qualitative findings revealed important insights that expanded the understanding from the quantitative strand, the integration would also incorporate complementary elements to ensure a richer, contextualized understanding. This iterative and integrative methodological approach allowed us to answer our overarching mixed-method research question comprehensively by drawing on both quantifiable patterns and lived experiences.

## Results

### Descriptive statistics

Table 1 presents characteristics of the 95 care recipients with cognitive impairment reported by their caregivers. Age ranged from 53 to 102 years (Mean = 74.4, *SD* = 13.5); the majority were aged 65 or older. The gender distribution was nearly balanced, with a slightly higher proportion of females (55.8%). Most care recipients were white (88.4%). Most care recipients lived with the respondent caregiver (61.05%) and lived in a one-family house (75.8%), with fewer residing in apartments (20.0%) or other housing types. Based on QDRS scores, most care recipients fell within the spectrum of dementia, with 55.8% having mild dementia and 24.2% having moderate-to-severe dementia. However, only 65.3% had received a formal diagnosis of Alzheimer’s disease or any type of dementia.

**Table 1:** Characteristics of care recipients with cognitive impairment (*N *= 95).

Characteristics	Frequency (**%**)
**Demographics**	
**Age (years)**	
50–64	29 (30.53)
65–84	39 (41.05)
85 or above	27 (28.42)
**Gender**	
Female	53 (55.79)
Male	42 (44.21)
**Race**	
White	84 (88.42)
Non-White	11 (11.58)
**Living conditions**	
**Co-residence with respondent caregiver**	58 (61.05)
**Housing type**	
A one-family house	72 (75.79)
Apartment	19 (20.00)
A mobile home or trailer	2 (2.11)
Others	2 (2.11)
**Cognitive condition and diagnosis status**	
**Severity of dementia rated with the QDRS**	
Normal^a^ to mild cognitive impairment	19 (20.00)
Mild dementia	53 (55.79)
Moderate-to-severe dementia	23 (24.21)
**Diagnosis status**	
Yes	62 (65.26)
No/don’t know	33 (34.74)

*Note*. QDRS = Quick Dementia Rating System.

a Although QDRS scores for the two care recipients fell within the normal range, both participants explicitly confirmed that the person they cared for had experienced a decline in memory or other thinking skills severe enough to impair daily functioning, including dementia and related disorders.

Neighborhood characteristics were summarized across four domains, each comprising a different number of detailed features (see Tables S1 and S2). Safety domain included 17 detailed items, with a mean score of 8.12 (*SD* = 7.59), indicating that most care recipients were reported to live in generally safe neighborhoods, characterized by features such as well-maintained streets, clear crosswalks, sufficient lighting, and the absence of crime and social disorder. Accessibility domain included 10 detailed items, with a mean score of 3.24 (*SD* = 6.06), suggesting many care recipients had walkable access to at least some key neighborhood destinations. Comfort domain included seven detailed items, with a mean score of 4.05 (*SD* = 3.48), indicating that many care recipients lived in neighborhoods perceived to offer a generally pleasant and comfortable sensory environment, such as natural features, quietness, and visual appeal. Legibility/Distinctiveness domain included six detailed items, with a mean score of 2.83 (*SD* = 3.22), reflecting modest support of care recipients’ neighborhoods for environmental cues (e.g., distinctive landmarks, readable signage, clearly defined public spaces) that aid in orientation and wayfinding.

### Quantitative results

Table 2 presents the multivariable logistic regression results examining whether dementia-friendly neighborhood features predict the AIP outcome and whether cognitive condition or dementia diagnosis modifies these associations. Four models were estimated to test the study hypotheses, progressively incorporating confounding variables and interaction terms. To examine whether symptom severity modifies neighborhood influences, Model 3 introduced interaction terms between neighborhood features and cognitive impairment severity and identified a significant interaction between neighborhood comfort and symptom severity. However, comfort significantly influenced the AIP outcome only among individuals with moderate-to-severe dementia, where the association was negative.

**Table 2:** Multivariable logistic regression results predicting AIP outcomes.

Variables	Main effects models		Interaction models	
	Model 1	Model 2	Model 3	Model 4
**Main variables**	**Odds ratios [95% CI]**			
Safety	1.04 [0.98, 1.11]	1.02 [0.95, 1.10]	1.06 [0.92, 1.21]	0.88* [0.77, 1.00]
Accessibility	1.00 [0.89, 1.12]	1.01 [0.86, 1.20]	1.05 [0.84, 1.32]	1.09 [0.84, 1.41]
Legibility and distinctiveness	0.89 [0.71, 1.11]	0.93 [0.71, 1.24]	0.79 [0.53, 1.17]	0.86 [0.52, 1.42]
Comfort	1.00 [0.86, 1.16]	0.94 [0.76, 1.15]	1.27 [0.93, 1.74]	0.77 [0.55, 1.08]
**Moderator variables**				
QDRS			4.40 [0.83, 23.23]	
Diagnosis				0.09* [0.01, 0.80]
**Interaction terms**				
QDRS × Safety			1.00 [0.88, 1.13]	
QDRS × Accessibility			0.97 [0.80, 1.17]	
QDRS × Legibility and distinctiveness			1.22 [0.88, 1.69]	
QDRS × Comfort			0.71*^,a^ [0.52, 0.95]	
Diagnosis × Safety				1.25** [1.08, 1.45]
Diagnosis × Accessibility				0.89 [0.64, 1.22]
Diagnosis × Legibility and distinctiveness				1.25 [0.67, 2.35]
Diagnosis × Comfort				1.17 [0.76, 1.80]
**Control variables included** ^b^	No	Yes	Yes	Yes
**Model statistics**				
Pseudo *R*^2^	0.02	0.13	0.18	0.22
Sample size (*N*)^2^	95	95	95	95

*Note*. AIP = aging in place; QDRS = Quick Dementia Rating System.

a As the main effect of comfort was non-significant while its interaction with symptom severity was significant, marginal effect estimation were conducted and indicated the significant association was confined to the moderate-to-severe dementia group (*p* < .05).

b To avoid overfitting with a relatively small sample size (*N *= 95), we tested a range of individual-level covariates and retained only those with substantive relevance and statistical contribution to the outcome. The final interaction models controlled for age (a continuous variable), gender (female or male), race (White vs. non-White), housing type (conventional vs. non-conventional housing), and co-residence with the respondent caregiver (yes or no). Additional robustness checks incorporating caregiving- and resource-related control variables are reported in Table S3.

*.01 ≤ *p* < .05.

**p < .01.

To examine whether dementia diagnosis moderates neighborhood influences, Model 4 tested interactions with diagnosis status. A significant interaction was observed between dementia diagnosis and neighborhood safety (*p* < .01). Among those with a formal diagnosis of Alzheimer’s disease or other dementias, neighborhood safety showed a positive association with the AIP outcome: each additional point on the safety scale, assigned for the presence of a positive safety feature (e.g., well-lit streets, clearly marked crosswalks) or for the absence of a negative safety feature (e.g., high crime rate, abandoned buildings), corresponded to an estimated 10% increase in the odds of AIP (combined OR = 1.10). In contrast, among individuals without a formal diagnosis of Alzheimer’s disease or related dementias, neighborhood safety was significantly associated with lower odds of AIP (OR = 0.88, *p* < .05).

### Qualitative results

Of the 95 caregivers surveyed, we obtained valid qualitative responses from 68 caregivers. Table 3 presents caregiver-reported neighborhood features identified as barriers or facilitators to AIP, along with representative direct quotes.

**Table 3:** Caregiver-reported neighborhood barriers/facilitators to AIP and representative quotes.

Environmental domain (Total mentions by caregivers)	Frequency of caregiver reports (*N* = 68)^a^		Representative quotes
	Cognitive condition• Normal to MCI (*N* = 18)• Mild dementia (*N* = 32)• Moderate-to-severe dementia (*N* = 18)	Diagnosis status• Diagnosis (*N* = 41)• Undiagnosed (*N* = 27)	
**Neighborhood built environment**			
**Safety (29)**	• Normal to MCI: 6 (33.33%) • Mild dementia: 17 (53.13%) • Moderate-to-severe dementia: 6 (33.33%)	• Diagnosis: 18 (43.90%) • Undiagnosed: 11 (40.74%)	• “Poorly maintained street surfaces and absence of sidewalks (make it difficult to age in place).” (C127) • “Her home is in a good neighborhood but it is a street with a pretty high speed limit. There are cross walks nearby, but not reliably all over.” (C123). • “Low crime (makes it easier to age in place).” (C029) • “Gated communities that allow them to walk around and get help if they get lost.” (C045)
**Accessibility (19)**	• Normal to MCI: 6 (33.33%) • Mild dementia: 9 (28.13%) • Moderate-to-severe dementia: 4 (22.22%)	• Diagnosis: 9 (21.95%) • Undiagnosed: 10 (37.04%)	• “Accessibility-flat sidewalks, hand rails, ramps as needed (makes it easier to age in place)” (C057) • “Resources such as grocery stores are within walking distance for most members of the community, but the routes to them are not direct, so it can be easy to get lost on the way there or back.” (C083) • “Our community is spread out so it can be hard to get around.” (C029)
**Comfort (13)**	• Normal to MCI: 6 (33.33%) • Mild dementia: 6 (18.75%) • Moderate-to-severe dementia: 1 (5.56%)	• Diagnosis: 7 (17.07%) • Undiagnosed: 6 (22.22%)	• “Noisy places make it difficult (to age in place).” (C088) • “Not always much shade—some trees for shade, but it gets hot … older people are more temperature-sensitive.” (C120) • “Beautiful environment to enjoy (for AIP).” (C073) • “Community greening (makes it easier to age in place).” (C672)
**Legibility and distinctiveness (12**)	• Normal to MCI: 4 (22.22%) • Mild dementia: 6 (18.75%) • Moderate-to-severe dementia: 2 (11.11%)	• Diagnosis: 4 (9.76%) • Undiagnosed: 8 (29.63%)	• “No landmarks or memorable places or markings (makes it difficult to age in place).” (C099) • “Lots of community markers also tend to look the same, so it can be hard to find your way even without memory problems.” (C083) • “Readable street signs (make it easier to age in place).” (C091)
**Familiarity (8)**	• Normal to MCI: 3 (16.67%) • Mild dementia: 2 (6.25%) • Moderate-to-severe dementia: 3 (16.67%)	• Diagnosis: 5 (12.20%) • Undiagnosed: 3 (11.11%)	• “Familiar surroundings (makes it easier to age in place).” (C103) • “It’s known to the person, so memory loss isn’t always as triggered as a new place (sometimes, anyway!).” (C120)
***Community and health services (23)***	• Normal to MCI: 5 (27.78%) • Mild dementia: 11 (34.38%) • Moderate-to-severe dementia: 7 (38.89%)	• Diagnosis : 17 (41.46%) • Undiagnosed: 6 (22.22%)	• “Social services and robust healthcare options (make it easier to age in place).” (C032) • “To spend so much time at home alone and not have any contact or help from other people such as help with meals and house work (making it difficult to age in place).” (C069) • “In-home care is needed (for AIP).” (C052)
***Respect and social inclusion (22)***	• Normal to MCI: 7 (38.89%) • Mild dementia: 13 (40.63%) • Moderate-to-severe dementia: 2 (11.11%)	• Diagnosis: 12 (29.27%) • Undiagnosed: 10 (37.04%)	• “Neighbors know and care for each other. Check on others if they have not been seen out of their routine.” (C100) • “No people from same age group (makes it difficult to age in place).” (C056) • “Friendly and helpful neighbors and pharmacy staff (make it easier to age in place).” (C071)
***Social participation (9)***	• Normal to MCI: 2 (11.11%) • Mild dementia: 5 (15.63%) • Moderate-to-severe dementia: 2 (11.11%)	• Diagnosis: 6 (14.63%) • Undiagnosed: 3 (11.11%)	• “Lack of senior center or other community (makes it difficult to age in place).” (C042) • “There is no place to take a person for the social interaction of day care.” (C055) • “… as much as possible for the elderly to participate in some social activities suitable for them.” (C296)
***Transportation (6)***	• Normal to MCI: 1 (5.56%) • Mild dementia: 1 (3.13%) • Moderate-to-severe dementia: 4 (22.22%)	• Diagnosis: 4 (9.76%) • Undiagnosed: 2 (7.41%)	• “Lack of transportation close to the home (makes it difficult to age in place). It is too far to walk.” (C070) • “Available, trained, convenient transportation (makes it easier to age in place).” (C042)

*Note*. AIP = aging in place; MCI = mild cognitive impairment.

a Among the 95 caregivers, 68 provided valid qualitative responses. Frequencies represent the percentage of caregivers in each subgroup who mentioned a given neighborhood feature within the environmental domain. Accordingly, percentages are calculated using the total number of caregivers in each subgroup as the denominator, not the total number who mentioned features within the domain. For example, although 29 caregivers mentioned safety concerns overall, 17 were caregivers of individuals with mild dementia (*n* = 32); therefore, the frequency for this subgroup is 53% (17/32).

Under the guidance of dementia-friendly design principles, qualitative coding identified five built environment domains raised by caregivers: safety (*n* = 29), accessibility (*n* = 19), comfort (*n* = 13), legibility/distinctiveness (*n* = 12), and familiarity (*n* = 8), as critical factors influencing AIP outcomes. Safety was clearly prioritized: for example, traffic-related concerns and the condition of pedestrian infrastructure were emphasized repeatedly by caregivers. Accessibility, including proximity to key destinations, was the second most cited domain, followed by comfort (e.g., neighborhood greenery) and legibility/distinctiveness (e.g., landmark, signage). Familiarity also emerged as an important theme as caregivers emphasized that remaining in one’s own home and being surrounded by recognizable, familiar environments made AIP easier, whereas new settings could trigger panic.

Comparisons by cognitive condition revealed that caregivers’ environmental priorities varied across dementia severity levels. Overall, the pattern suggests that the perceived influence of neighborhood environments on AIP did not increase progressively with dementia severity. Caregivers’ emphasis on most built environment domains (accessibility, comfort, and legibility/distinctiveness) demonstrated an inverse relationship with cognitive impairment severity, that is, the proportion of caregivers prioritizing these features progressively declining across cognitive status groups. For example, in the domain of comfort, 33% (6 out of 18) of caregivers for individuals with normal cognition or MCI mentioned it as important, compared to 19% (6 out of 32) in the mild dementia group, and only 6% (1 out of 18) in the moderate-to-severe dementia group. Safety was prominent among caregivers of individuals with mild dementia, while familiarity appeared less important within this same group. For caregivers of individuals with moderate-to-severe dementia, comfort was relatively less emphasized, whereas safety and accessibility remained among the more important considerations.

On the other hand, comparisons by diagnosis status highlighted notable differences regarding the perceived importance of certain domains. Caregivers in both groups consistently emphasized safety and familiarity as important considerations for AIP, with those caring for individuals diagnosed with dementia placing slightly greater emphasis on these factors. However, caregivers of individuals without a formal diagnosis placed greater emphasis on accessibility and legibility/distinctiveness, with these domains mentioned two to three times more often than by caregivers of those with a diagnosis.

Caregivers also frequently emphasized other domains that were less directly related to the built environment, particularly community and health services (*n* = 23) and respect and social inclusion (*n* = 22), indicating their importance was comparable to built environment domains such as safety. Notably, a greater emphasis on community and health services was associated with dementia severity. Transportation-related issues were highlighted significantly more often by caregivers of individuals with moderate-to-severe dementia, while respect and social inclusion received relatively fewer mentions within this group. Comparisons by diagnosis status also revealed distinct priorities: caregivers of individuals with dementia diagnoses emphasized community and health services, social participation, and transportation, whereas caregivers of individuals without diagnoses placed greater focus on respect and social inclusion.

### Integrated results for meta-inference

Both quantitative and qualitative strands highlighted safety as a central neighborhood built environment factor influencing AIP outcomes. The quantitative analysis revealed a highly significant interaction: among individuals with a formal dementia diagnosis, higher perceived neighborhood safety (e.g., well-maintained streets, low crime rates, manageable traffic volumes and speeds, sufficient pedestrian infrastructure) was associated with increased odds of AIP. Consistent with this, qualitative findings revealed that caregivers frequently and explicitly emphasized neighborhood safety, particularly those caring for individuals with a formal dementia diagnosis. Caregivers’ narratives featured concerns around traffic conditions and pedestrian infrastructure, emphasizing their direct impact on remaining in the community.

Another convergence revealed that, with both strands, the built environment’s impact on AIP outcomes was limited and not substantially moderated by dementia severity. In quantitative results, only the moderate-to-severe dementia group showed a significant negative effect of comfort. Qualitative findings showed that caregiver emphasis on some environmental features even declined as impairment worsened, which may be partially due to the fact that people with significant cognitive impairment go out less often.

Despite broad convergence on the importance of safety, nuanced differences emerged. In quantitative results, perceived safety was associated with reduced odds of AIP among individuals without a dementia diagnosis. In contrast, qualitative findings portrayed safety as an important factor for both diagnosed and undiagnosed individuals. Further divergence appeared that accessibility, comfort, and legibility/distinctiveness were not statistically significant predictors of the AIP outcome in the quantitative model, but caregivers’ narratives highlighted their practical importance. Accessibility (e.g., proximity to essential services and recreational spaces, physical accessibility) emerged consistently as critical across groups with and without a diagnosis. Caregivers of diagnosed older adults frequently mentioned comfort (e.g., quietness, greenery, and aesthetic appeal) as particularly supportive. Conversely, caregivers of individuals without a formal diagnosis placed greater emphasis on legibility/distinctiveness, including identifiable landmarks, understandable signage, and environmental cues.

Environment domains less directly related to the built environment showed important influences on the AIP outcome in qualitative data, although they were not examined in quantitative analyses. Community and health services (e.g., availability of professional home care, dementia-specific programs, day services) and respect and social inclusion (e.g., active support and monitoring from neighbors, community dementia-awareness) emerged as highly influential according to caregivers’ lived experiences in facilitating AIP.

**Figure 2:**
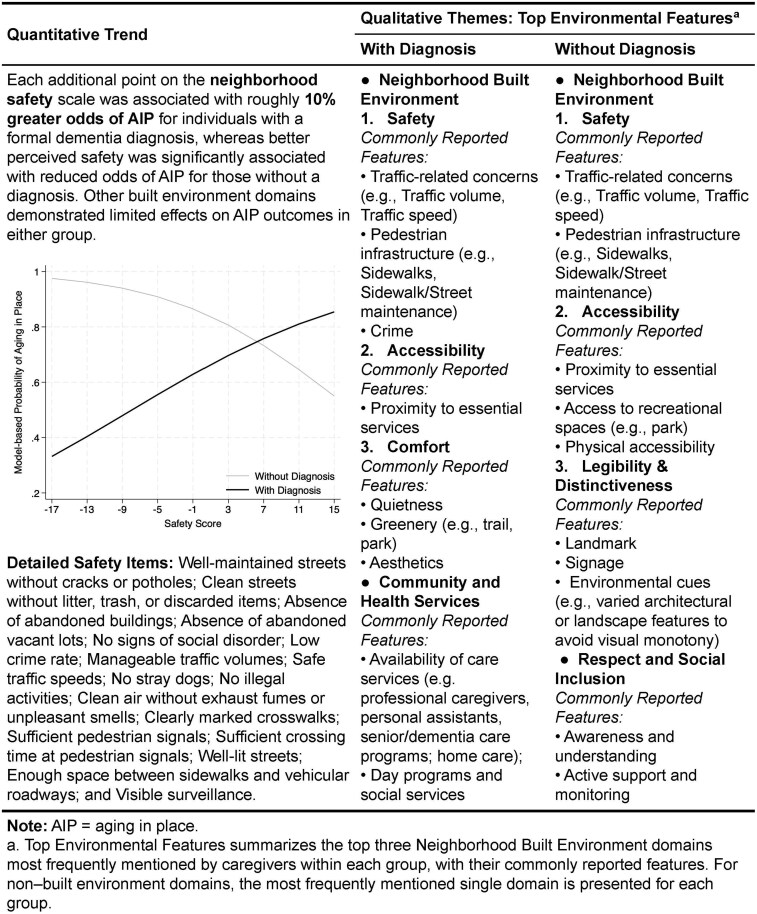
Joint display of quantitative and qualitative results: neighborhood environmental domains by diagnosis status.

Given the limited influence of impairment severity, Figure 2 reports the results stratified by dementia diagnosis. The meta-inference suggests that neighborhood built environments significantly influenced AIP outcomes, but caregiver perceptions of these influences varied notably by dementia diagnosis and across environmental domains. Safety emerged consistently as a central domain. However, diagnosis status moderated how safety influenced AIP decisions, highlighting the critical role of dementia diagnosis in caregivers’ environmental perceptions. Qualitative findings enriched quantitative results by caregivers’ lived experiences, emphasizing additional built environment characteristics (e.g., accessible destinations), health services, and community supports.

## Discussion and implications

This study addressed critical gaps in the existing literature regarding the potential roles of neighborhood built environments on AIP outcomes among older adults with cognitive impairment. It further examined how the severity of cognitive impairment and dementia diagnosis shape environmental perceptions and AIP decisions. Our findings underscored the important role of neighborhood safety, particularly emphasizing how diagnostic status moderated caregivers’ perceptions and environmental responses. Specifically, we found each additional point on the neighborhood safety scale was associated with roughly 10% greater odds of AIP for individuals with a formal dementia diagnosis. Caregivers’ qualitative narratives further underscored safety and accessibility as critical determinants of AIP decisions. These findings align with findings from studies on general aging populations (Choi, 2022; Ratnayake et al., 2022).

Drawing on the Ecology of Aging and the Person–Environment Competence model (Lawton, 1982; Lawton & Nahemow, 1973), the built environment may shape AIP outcomes by influencing behavioral responses to everyday settings, a dynamic interplay of environmental press and individual competence. AIP poses distinct behavioral challenges, including safety risks (e.g., fall risks, wandering), increased caregiver burdens (e.g., high caregiving responsibility), and reduced autonomy (e.g., impaired self-care, disrupted daily routines) (Thoma-Lürken, 2018). The environmental features (e.g., pedestrian safety, service accessibility), thus, may operate through both push and pull mechanisms in relocation decisions (Forsyth & Molinsky, 2021). Safety concerns may act as push factors prompting families to consider relocation, while proximity to caregivers or better service access may function as pull factors drawing them toward more supportive settings. Viewed this way, AIP reflects the broader need for person–environment fit, which underpins the ecological perspective guiding this study. While our study focused on a traditional form of AIP within private residences, the push–pull dynamics made AIP not solely about AIP but about aging in the right place. The role of the built environment is not limited to preserving continuity but also includes enabling adaptive transitions across diverse settings.

Consistent with an ecological view, our findings also highlight the importance of diagnostic status in shaping caregivers’ environmental responses, revealing a pathway from diagnosis to adaptive behaviors. Prior research has demonstrated that diagnosis typically triggers behaviors, including actively seeking help and developing tailored coping strategies to address intensifying care demands (Stokes et al., 2015). A formal diagnosis can also transform caregivers’ perception and responses to their neighborhood environment. They may become more attuned to barriers or facilitators relevant to residential decisions, which may enable more effective caregiving, strengthen coping capacity, and help delay institutional transitions, thereby supporting AIP. While diagnosis can trigger adaptive responses, it may also reflect underlying contextual advantages, as access to timely evaluation and care planning often depends on financial and service resources. Such advantages may coincide with residence in better-resourced environments, shaping AIP trajectories through enhanced support and environmental fit.

Our study also uncovered unexpected insights into how the neighborhood built environment influences AIP. Accessibility and legibility/distinctiveness did not significantly predict the AIP outcome, nor did dementia severity or diagnosis status modify these relationships. The qualitative findings further echoed this trend—caregivers’ emphasis on these domains declined as cognitive impairment severity increased. This may be because advancing cognitive impairment typically leads to restricted activity spaces in indoor environments (Kaye et al., 2011; Shoval et al., 2011), diminishing caregivers’ concerns for outdoor neighborhood environments. Furthermore, individuals with dementia tend to attribute their challenges to personal failings rather than environmental inadequacies (Bartlett & Brannelly, 2019; Biglieri & Dean, 2021). Such mindsets may carry over to caregivers (Marhánková & Honelová, 2024), leading them to deemphasize the role of environmental conditions in AIP regardless of neighborhood quality. We also found familiarity was the least frequently mentioned environmental domain in our qualitative findings, although aging in a familiar environment is highlighted in current literature (Bigonnesse & Chaudhury, 2020). A nationwide expert survey questioned the dominant focus on familiarity in dementia-friendly discourse, echoing our findings (Chen et al., 2025). Future research should investigate these complex dynamics using larger samples and/or deeper lived-experience approaches.

Another unexpected finding was that higher perceived safety was associated with lower odds of AIP among those without a diagnosis. One possible explanation is a mismatch between current perceptions and future-oriented housing decisions. Individuals without a formal diagnosis may remain relatively more active and thus have greater exposure to their neighborhood, which can increase awareness of neighborhood issues and future risks (Jack & McCormack, 2014). In this context, higher perceived safety may reflect current manageability rather than long-term suitability and may coexist with more proactive planning to relocate as needs increase (Lawton, 1989). Prior work likewise shows that perceived and objective environments often diverge and that perceptions can shape behaviors in complex ways (Gebel et al., 2009).

Similarly, higher perceived comfort predicted lower odds among those with moderate-to-severe dementia, while qualitative data showed that this group placed the lowest priority on neighborhood comfort. This pattern suggests that comfort may be less salient for AIP decisions at more advanced stages. As competence declines, environmental press and care demands become more consequential, such that safety, supervision, and support availability may outweigh experiential qualities such as comfort (Lawton & Nahemow, 1973). Furthermore, the measures we used may reflect not only the conditions of the neighborhood themselves but also individual resources, exposure, and other unobserved factors that may influence outcomes simultaneously. Future studies with larger samples would help test these mechanisms more directly and to refine environmental measurements that are more specific and tailored for older adults with cognitive impairment.

It is important to recognize several limitations of this study. First, our relatively small sample size and limited qualitative responses reduced the statistical power and the depth of qualitative insights, reducing our ability to discern nuanced associations between neighborhood environments and AIP outcomes. It also limited our ability to fully account for potential confounders that may shape these relationships. Second, data collection during COVID-19 introduced potential selection bias. Exclusive online recruitment due to COVID-19 protocols may have biased our sample toward more technologically proficient caregivers with more connections to support networks. Additionally, despite instructions to report on pre-pandemic conditions, heightened environmental awareness during COVID-19 may have influenced participants’ retrospective perceptions. Third, due to limited capacity for valid in-depth data from individuals with cognitive impairment, we relied on caregiver reports, which may introduce recall bias and misreporting. This is especially relevant for neighborhood perceptions, which can be highly personal and situational. Although some domains (e.g., safety, accessibility) may be more readily observed, others (e.g., familiarity, comfort) often require first-person lived experience to be captured accurately. Meanwhile, caregivers were instructed to report based on the care recipient’s anticipated relocation intentions; however, proxy reports may still reflect caregivers’ own interpretations, priorities, and constraints, as well as household dynamics and resource availability. Future studies may benefit from participatory methods (e.g., walking interviews, photovoice, or digital diaries) to obtain authentic, real-time neighborhood experiences when objective measures are difficult to obtain.

Despite these limitations, our study provides important insights into the interplay of neighborhood environments, diagnostic status, and AIP decisions among older adults with cognitive impairment and highlights key areas for ongoing research and interventions. Employing a convergent mixed-methods design enabled an integrated analysis, capturing both statistical trends and lived experiences. Future research may also consider sequential approaches to more strategically leverage the distinct strengths of different methods. A stepwise design may facilitate the identification of key themes or subgroups through one strand. This can then be systematically examined or extended in the other, moving beyond corroboration to enable deeper merging and complementary insights. Additionally, future studies would benefit from larger, more diverse samples with longitudinal and multi-informant designs. This may offer stronger evidence on how environmental factors relate to AIP outcomes over time, and to better separate these associations from other potentially correlated factors such as caregiving context and household resources. Meanwhile, participatory methods can also be effective tools to directly engage and elicit meaningful subjective data from those with cognitive decline (Sturge et al., 2021). Integrating objective environment measures (e.g., Geographic Information System, environmental audit tools) can also provide a more comprehensive understanding of environmental barriers and facilitators.

Our findings underscore the need for holistic dementia-friendly neighborhood interventions that extend beyond physical design alone. Timely diagnosis may shape caregivers’ awareness and prioritization of the built environment, while community awareness and coordinated service supports are also important for meaningful AIP. Creating adaptive neighborhood ecosystems can support AIP across the dementia trajectory by integrating clinical understanding of disease progression with environmental design responsive to evolving needs.

While this study adopts a place-based operationalization of AIP and is grounded in an ecological theoretical framework, future research could explore how the alternative definitions and theoretical perspectives, such as those emphasizing autonomy, control, or life-course transitions, shape the interpretation of neighborhood influences, especially in cognitively diverse aging populations. Supporting AIP for this growing population requires greater diagnostic and environmental awareness, along with cross-sector collaboration among built environment professionals, healthcare providers, and community stakeholders. These efforts can reduce institutional care costs and ease caregiver burden.

## Conclusion

This study examined how neighborhood built environments and cognitive conditions influence AIP outcomes for older adults with cognitive impairment. We found that most built environment features showed limited associations with the AIP outcome, regardless of dementia severity. However, dementia diagnosis status plays an important moderating role, with each increment on the safety scale associated with greater odds of AIP among those with a formal diagnosis. Caregivers’ narratives from the qualitative analyses reinforced these quantitative results, emphasizing safety as a critical determinant alongside access to neighborhood destinations and community services and support. Our findings underscore the important role of dementia diagnosis in shaping environmental responsiveness, revealing that formal diagnosis fundamentally alters how caregivers perceive and prioritize neighborhood characteristics. These insights suggest that effective AIP interventions should integrate timely diagnosis processes with dementia-friendly design and robust community support networks. There is an urgent need for coordinated, cross-sector strategies to foster safe, accessible, and inclusive neighborhoods that support AIP for older adults with cognitive impairment more self-sufficiently and adaptively while reducing caregiver burden and reliance on institutional care.

## Supplementary Material

gbag057_Supplementary_Data

## Data Availability

The research data, coding, and research materials utilized in this study are available on a secured server that the primary researcher has exclusive access to. The raw data can be shared upon reasonable request. This study was not preregistered.
