# Supplementary material for: After diagnosis, place matters: the role of neighborhood built environment in aging in place among older adults with cognitive impairment
Source: J Gerontol B Psychol Sci Soc Sci. 2026 Apr 3;81(5):gbag057. doi: 10.1093/geronb/gbag057 (PMC13134383; doi:10.1093/geronb/gbag057)
Supplement: gbag057_Supplementary_Data [file gbag057_supplementary_data.pdf]

The Journals of Gerontology, Series B: Psychological Sciences and Social Sciences

Supplementary Material: Wang, Lee, Chen, Zhu, & Ory. After diagnosis, place matters: The role of neighborhood built environment in aging in place among older adults with cognitive impairment.

Supplementary Table 1. Neighborhood Environmental Survey Items Categorized by Dementia-Friendly Domains[a]

Domain	Detailed Neighborhood Characteristics[b]
Safety	Well-maintained streets without cracks, potholes
	Clean streets without litter, trash, or discarded items
	*Many abandoned buildings
	*Many abandoned vacant lots
	*Signs of social disorder
	*High crime rate
	*Too much traffic
	*Cars going too fast
	*Stray dogs
	*Illegal activities
	*A lot of exhaust fumes or unpleasant smells in the air
	Clearly marked crosswalks
	Sufficient pedestrian signals
	Sufficient crossing time at pedestrian signals
	Well-lit streets
	Enough space between sidewalks and vehicular roadways
	Visual surveillance
Accessibility	Stores (e.g. supermarkets, drug store, big box stores such as Target and Walmart)
	Services (e.g. restaurants, banks, postal services, hair salons)
	Parks and recreational facilities
	Other green spaces and gardens
	Public buildings (e.g. community center, library, civic building, religious institution)
	Public restrooms
	Neighborhood places where older adults can interact with each other
	Neighborhood places where older adults can interact with others of different ages
	Benches, outdoor seating, or resting areas
	Places to go for entertainment (e.g. theater, bar)
Comfort	Trees to provide shade along the streets
	Enough privacy
	Enough quietness
	Many attractive buildings, homes, or gardens
	Many beautiful natural features
	Pleasant wildlife to look at
	Pleasant sounds of nature
Legibility and Distinctiveness	Streets with distinctive landmarks
	Simple and clear signs
	Public buildings with clearly visible and identifiable entrances
	Street amenities (e.g. benches and tables) that have a variety of styles, materials, and colors
	Buildings and outdoor spaces that are easy to understand in terms of their purposes
	Traffic signs that are easy to read

[a] The domain of "Familiarity," part of the dementia-friendly framework, was excluded due to its highly subjective nature and limited measurability through caregiver reports.
[b] An asterisk (*) indicates a negative environmental characteristic.

Supplementary Table 2. Descriptive Statistics of Neighborhood Environmental Domains

Environmental Domains[a] (Cronbach's α[b])	Mean	SD	Median	Min	Max
Safety (0.85)	8.12	7.59	9	-13	17
Accessibility (0.86)	3.24	6.06	4	-10	10
Comfort (0.74)	4.05	3.48	5	-7	7
Legibility & Distinctiveness (0.73)	2.83	3.22	4	-6	6

[a] Detailed neighborhood characteristics included in each domain are provided in Supplementary Table 1.
[b] Internal consistency of multi-item scales was assessed using Cronbach's alpha (Cronbach's α ≥ .90 = Excellent, 0.80–0.89 = Good, 0.70–0.79 = Acceptable, 0.60–0.69 = Questionable, 0.50–0.59 = Poor, < .50 = Unacceptable).

Supplementary Table 3. Robustness check for additional confounders suggested by reviewer

Variables	Original Specification[a]	+ Community & Health Resources[b]	+ Caregiver Relationship Type[c]	+ Income[d]
		Model 3		
**Main Variables**				
Safety	1.06 [0.92, 1.21]	1.08 [0.93, 1.25]	1.07 [0.92, 1.24]	1.07 [0.92, 1.23]
Accessibility	1.05 [0.84, 1.32]	1.09 [0.84, 1.40]	1.07 [0.84, 1.37]	1.06 [0.85, 1.32]
Legibility & Distinctiveness	0.79 [0.53, 1.17]	0.96 [0.62, 1.48]	0.76 [0.49, 1.17]	0.77 [0.52, 1.13]
Comfort	1.27 [0.93, 1.74]	1.36 [1.00, 1.86]	1.23 [0.88, 1.72]	1.29 [0.93, 1.77]
**Moderator Variables:** QDRS	4.40 [0.83, 23.23]	6.63* [1.16, 37.96]	3.98 [0.73, 21.66]	4.75[0.88, 25.78]
**Interaction Terms**				
QDRS × Safety	1.00 [0.88, 1.13]	0.99 [0.86, 1.12]	0.99 [0.87, 1.12]	0.99 [0.88, 1.12]
QDRS × Accessibility	0.97 [0.80, 1.17]	0.99 [0.78, 1.25]	0.96 [0.79, 1.17]	0.96 [0.79, 1.17]
QDRS × Legibility & Distinctiveness	1.22 [0.88, 1.69]	1.19 [0.83, 1.72]	1.23 [0.87, 1.76]	1.24 [0.90, 1.71]
QDRS × Comfort	0.71* [0.52, 0.95]	0.67* [0.48, 0.93]	0.72* [0.53, 0.98]	0.70* [0.52, 0.95]
**Pseudo R2**	0.18	0.23	0.19	0.18
**Sample Size (N)2**	95	95	95	95
		Model 4		
**Main Variables**				
Safety	0.88* [0.77, 1.00]	0.88 [0.77, 1.01]	0.88 [0.77, 1.00]	0.87* [0.76, 0.99]
Accessibility	1.09 [0.84, 1.41]	1.10 [0.86, 1.40]	1.08 [0.83, 1.43]	1.08 [0.84, 1.37]
Legibility & Distinctiveness	0.86 [0.52, 1.42]	1.03 [0.56, 1.88]	0.86 [0.49, 1.52]	0.87 [0.55, 1.40]
Comfort	0.77 [0.55, 1.08]	0.80 [0.60, 1.07]	0.74 [0.51, 1.07]	0.76 [0.53, 1.10]
**Moderator Variables:** Diagnosis	0.09* [0.01, 0.80]	0.13 [0.02, 1.00]	0.09* [0.01, 0.90]	0.09* [0.01, 0.91]
**Interaction Terms**				
Diagnosis × Safety	1.25** [1.08, 1.45]	1.24** [1.07, 1.43]	1.25** [1.07, 1.44]	1.25** [1.07, 1.46]
Diagnosis × Accessibility	0.89 [0.64, 1.22]	0.94 [0.69, 1.29]	0.91 [0.66, 1.25]	0.90 [0.66, 1.24]
Diagnosis × Legibility & Distinctiveness	1.25 [0.67, 2.35]	1.19 [0.64, 2.24]	1.19 [0.61, 2.32]	1.23 [0.67, 2.25]
Diagnosis × Comfort	1.17 [0.76, 1.80]	1.10 [0.75, 1.64]	1.21 [0.78, 1.89]	1.19 [0.75, 1.88]
**Pseudo R^2**	0.22	0.25	0.22	0.23
**Sample Size (N)**	95	95	95	95

Notes. Because the sample size is modest and adding many covariates may risk overfitting, we retain the original specification from the main text and conduct the following targeted robustness checks. **a:** the original specification corresponds to Model 3 & 4 in the main text without any modifications. **b:** adjusts for a composite measure of Community and Health Services, coded using the same method as built-environment variables. This measure includes local health/supportive services, home care services, affordable and high-quality home health providers, wellness programs, caregiver training, fitness activities for older adults, and multilingual health professionals. **c:** adjusts for caregiver relationship type, grouped into spouse/partner/parent; child/child-in-law/grandchild; and other caregivers. Two dummies were included, with "other" as the reference. **d:** adjusts for household income, collapsed from the original categorical variable into a median-split (low vs. high). "Don't know/Prefer not to answer" responses form an implicit reference category. All models include the same covariates as the original specification.
